# Hierarchical self-entangled carbon nanotube tube networks

**DOI:** 10.1038/s41467-017-01324-7

**Published:** 2017-10-31

**Authors:** Fabian Schütt, Stefano Signetti, Helge Krüger, Sarah Röder, Daria Smazna, Sören Kaps, Stanislav N. Gorb, Yogendra Kumar Mishra, Nicola M. Pugno, Rainer Adelung

**Affiliations:** 1 Functional Nanomaterials, Institute for Materials Science, Kiel University, Kaiserstr. 2, 24143 Kiel, Germany; 2 Laboratory of Bio-Inspired and Graphene Nanomechanics, Department of Civil, Environmental and Mechanical Engineering, University of Trento, via Mesiano 77, I-38123 Trento, Italy; 3 Functional Morphology and Biomechanics, Zoological Institute, Kiel University, Am Botanischen Garten 1-9, 24098 Kiel, Germany; 4 Ket Lab, Edoardo Amaldi Foundation, Italian Space Agency, Via del Politecnico snc, 00133 Rome, Italy; 5 School of Engineering and Materials Science, Queen Mary University of London, Mile End Road, London, E1 4NS UK

## Abstract

Three-dimensional (3D) assemblies based on carbon nanomaterials still lag behind their individual one-dimensional building blocks in terms of mechanical and electrical properties. Here we demonstrate a simple strategy for the fabrication of an open porous 3D self-organized double-hierarchical carbon nanotube tube structure with properties advantageous to those existing so far. Even though no additional crosslinking exists between the individual nanotubes, a high reinforcement effect in compression and tensile characteristics is achieved by the formation of self-entangled carbon nanotube (CNT) networks in all three dimensions, employing the CNTs in their high tensile properties. Additionally, the tubular structure causes a self-enhancing effect in conductivity when employed in a 3D stretchable conductor, together with a high conductivity at low CNT concentrations. This strategy allows for an easy combination of different kinds of low-dimensional nanomaterials in a tube-shaped 3D structure, enabling the fabrication of multifunctional inorganic-carbon-polymer hybrid 3D materials.

## Abstract (web summary)

Low-dimensional nanomaterials are crucial conducting components of stretchable electronics, but their mechanical reinforcement remains challenging. Here, the authors infiltrate carbon nanotubes into a porous ceramic network to produce a 3D nanofelted self-entangled assembly with high conductivity and mechanical stability.

## Introduction

Incorporating the unique and exceptional properties of low-dimensional carbon materials like carbon nanotubes (CNTs) into macroscopic functional materials is still very challenging, and this is the reason why their day-to-day application remains limited to the laboratory scale. Nevertheless, CNTs are well known for their high electrical, thermal, optical as well as mechanical properties and thus they are one of the most widely studied materials within the last couple of decades. Different CNT-based assemblies have been reported, including one-dimensional (1D) fibers^1–4^, 2D films^5–7^ buckypapers^8–10^, and 3D aerogels^11–15^. The latter ones have opened many possibilities for new applications in the field of sensors^16,17^, catalysis^18–20^, supercapacitors^21,22^, as well as free-standing electrodes^23^, and recyclable absorbers^18,24,25^ mainly due to their extremely large surface to volume ratio, hierarchical porous structural morphology and, most important, the macroscopic dimensions^26^.

The decisive problem with CNT-based composites with respect to polymer reinforcement is the matrix-filler interaction. Without an effective chemical modification of the CNTs the externally applied stresses to the composite are not directly transferred to the tubes and thus no effective reinforcement takes place. However, the chemical modifications often weaken the structure of the CNTs^9^. Hence, a reliable reinforcement can only be achieved with ultra-long unmodified CNTs, which could be done either by welding, knotting, or felting of the individual tubes. The latter can already be realized in the form of 2D buckypapers infiltrated with polymers, leading to an increase in the mechanical properties by a factor of about 3, 9, and 28 with respect to modulus, strength, and toughness, respectively^9^. However, buckypaper-reinforced polymers with properties beyond 2D (films/foils/layers) are still missing. A realization of this approach in form of a real 3D composite material is shown here for the first time, to the best of our literature knowledge, leading to unique characteristics in mechanical as well as electrical properties of the stand-alone carbon material and of the resulting composites.

In general, the preparation methods of such 3D CNT networks can be divided into two classes: a dry chemistry (direct growth of CNTs using a chemical vapor deposition process (CVD)) or wet chemistry approach (CNT networks from a CNTs dispersion). The 3D CNT assemblies grown by CVD process show a high mechanical flexibility as well as a high electrical conductivity due to their long individual CNTs^13,14^, whereas the wet chemistry approaches offer well-controlled reaction conditions, tunable composition, and desired scalability^11,12,25,26^. However, both of these processes suffer from certain drawbacks, such as the necessity for a large amount of metal catalyst and the reliability of the reactions involved within the CVD process (e.g., control of the number of stacked, rolled up graphene sheets per CNT)^26,27^. As far as the wet chemistry approaches are concerned, the characteristics of 3D CNT assemblies depend mainly on the quality of the CNT dispersion, leading to many defects and shortening of CNTs resulting from the required ultrasonication steps^11,26,28^. There have also been several reports on the fabrication of 3D carbon structures derived from porous templates. These offer the possibility to form open pore structures, with pore sizes up to several microns in diameter. This allows for a tailorable pore-size distribution of macro- or meso-pores, in comparison to the closed pore structures (with pores mostly in the nanometer range) prepared by most wet-chemistry approaches^16,29–32^. For most of the applications, the open pore structures, with pores on the micrometer scale, are highly beneficial, since they allow for a large and especially highly accessible surface area (especially for nanostructured pore surfaces), which is not the case for closed pore structures. However, those template-based methods, which always rely on metal foams (mainly nickel), are based on the direct growth of CNTs by means of CVD and thereby creating not only CNTs but also CNT/graphene composites due to the overlapping growth conditions^26,27^.

Macroscopically expanded (>cm^3^ size) and highly porous 3D CNT tube (CNTT) networks are realized by a direct wet chemical infiltration of dispersed CNTs into a porous ceramic network^33^, thereby overcoming most of the drawbacks of other well-known synthesis methods^26,27^. The infiltrated CNTs form self-entangled networks surrounding the ceramic network in the form of a nanofelted assembly, resulting in a double-hierarchical CNTT architecture. The concept enables the formation of open porous CNTT stand-alone networks, mechanical reinforced and conductive CNTT/ceramic, and CNTT/polymer composites. The highly porous (~ 93%) ceramic becomes significantly reinforced under both tensile (factor of ~ 40) and compression (factor of ~ 147) loadings when infiltrated with self-entangled CNTs, being able to support more than 10^5^ times their own weight (with length of ~ 6 mm, porosity >87%). Finite element method (FEM) simulations confirm that in such a hybrid structure, the CNTs are employed in their extraordinary tensile properties, even though the structure is loaded in compression (similar to tensegrity). To the best of our knowledge, such high reinforcement gain has never been shown by any macroscopic 3D CNT composite structure prepared by wet chemical means. The CNTT assemblies show high conductivity (~130 S m^−1^) at low densities and a higher mechanical stability (~5 times, ultimate compressive strength ~0.25 MPa) in contrast to conventional 3D CNT assemblies^11,13,26,34,35^, due to their self-entangling nature. Additionally, the double-hierarchical structure allows the decrease of the percolation threshold in polymers and enables self-enhancing stretchable conductors. A 3D CNTT structure has been realized here for the first time and it exhibits mechanical and electrical properties suitable for a wide range of technologies, including stretchable conductors, gas sensing, cell-scaffold materials, and cathode materials for batteries. In contrast to most other 3D nanocarbon architectures^11,13,14,26,34^, the structure presented here is an open pore structure with a high porosity and pores in the range of several micrometers, which is beneficial for several applications due to the ease in high surface accessibility.

## Results

### 3D CNTT/ceramic composites

**Fig. 1:**
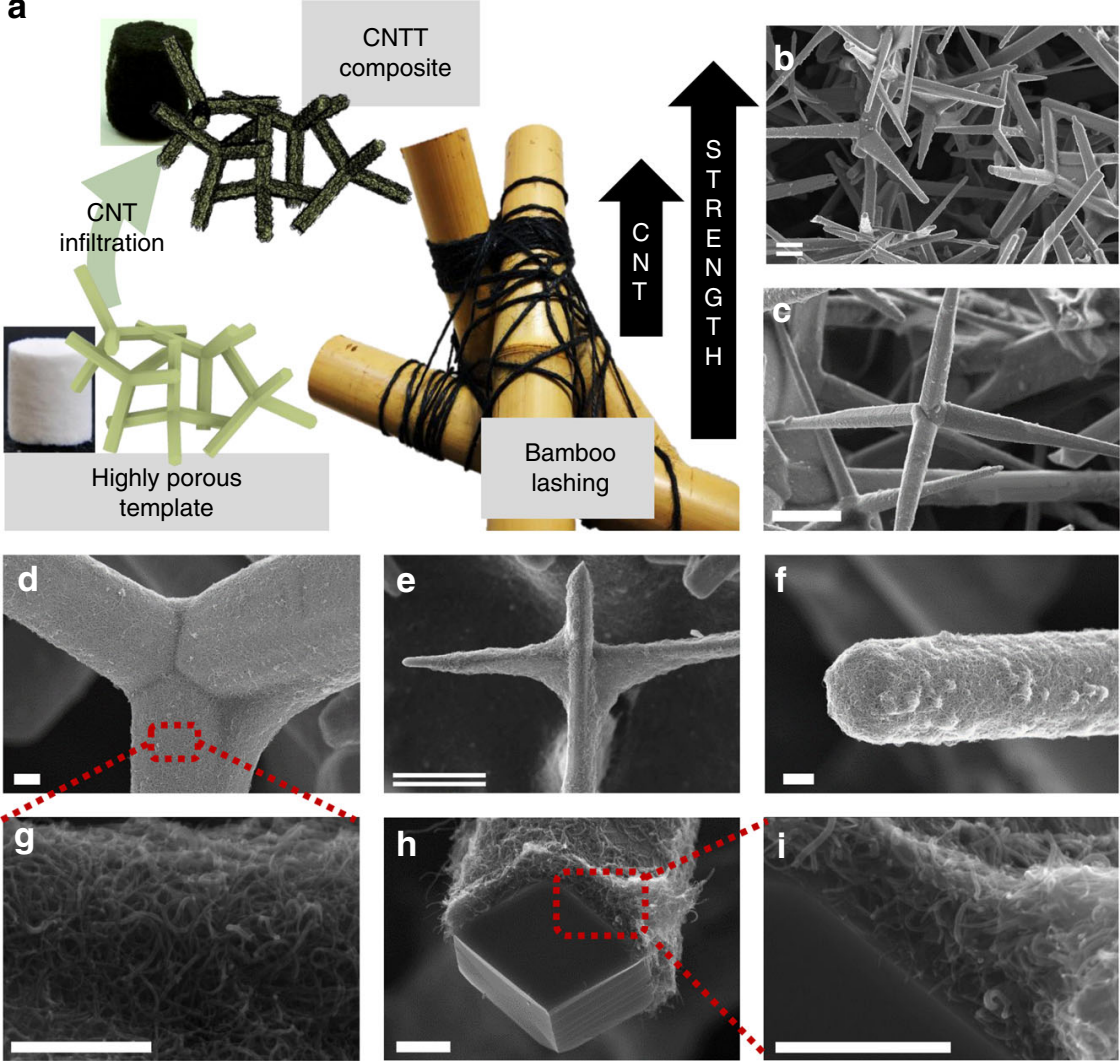
Overview of the CNTT/t-ZnO composite structure. **a** Schematic representation of the fabrication process showing the highly porous (93%) ceramic network consisting of pre-sintered tetrapodal shaped ZnO microparticles (*V* = 1.7 cm^3^) before and after CNT infiltration. By adding CNTs to the network self-entangled CNT networks are formed leading to a high mechanical reinforcement effect, similar to traditional bamboo lashing; **b**, **c** SEM images of the ceramic network coated with a homogenous layer of self-entangled carbon nanotubes; **d** Detail of the CNT network around ZnO tetrapod central joint; **e** Detail of CNT network around tetrapods junction; **f**, **g** High magnification SEM images showing that the CNTs form self-entangled layers on the ceramic template; **h**, **i** Self-entangled CNT layer on a broken tetrapod arm, revealing the thickness of the coated CNT layer. (Scale bars: single: 1 µm, double: 10 µm)

Fabrication of the 3D composite structures, consisting of CNTs on a ceramic template of a porous interconnected network of ZnO tetrapods (CNTT/t-ZnO), was achieved by a simple dripping method, as illustrated in Fig. 1a. Heated cylindrical pre-sintered highly porous ceramic t-ZnO networks^32,36^ (Supplementary Fig. 1) with a low density of 0.3 g cm^−3^ (porosity >93%) were infiltrated with a commercially available aqueous CNT dispersion using a custom designed computer controlled syringe and dried under ambient conditions (see Supplementary Movie 1). The amount of CNTs was controlled precisely by repeated infiltrations of the template. Representative scanning electron microscopy (SEM) images of the structural morphologies are shown in Fig. 1b–i, revealing the successful coverage of the ceramic 3D network with interwoven layers of self-entangled CNTs.

The detailed SEM study suggests that almost the entire porous network is covered with a homogeneous felted CNT layer (Supplementary Fig. 2) with a thickness ranging from the nano to the submicron scale. The prerequisites for a uniform distribution of CNTs are a highly porous 3D ZnO (as porous as possible, in the present case of ~93% porosity) with a hydrophilic surface^37,38^, as well as the high concentration and dispersion quality of the used CNT ink. It is important to note that these templates have open pores of several µm in diameter. Only thereby is a homogeneous coating possible. A more detailed discussion of the requirements of the template as well as the CNT dispersion etc., can be found in the Supplementary Discussion section. The high porosity of the ceramic leads to large capillary forces upon contact with the CNT ink allowing the liquid to be easily infiltrated and rapidly absorbed. Accordingly, due to the hydrophilic nature of the ceramic network, the aqueous dispersion will maximize its interface area upon CNT infiltration process. Increasing the amount of infiltrated dispersion will increase the wetted interface area and thus the infiltrated volume, up to a point where the entire template is filled with the CNT dispersion (as seen in Supplementary Movie 1). Due to the high porosity of the network no self-filtering effect can be observed, even when the template is already infiltrated with a high CNT load. For the formation of the self-entangled CNT layer, two fundamental mechanisms could be most likely responsible. First, upon infiltration, some CNTs will directly attach to the ZnO surface due to its high polarization and thus particle-substrate force (Supplementary Movies 2 and 4). Second, during drying, the remaining CNTs are deposited on the template by the formation of water menisci between the ZnO junctions, similar to the processes in dip coating or convective self-assembly^39–41^. Surfaces that are already covered with CNTs will barely attach further CNTs due to the stabilizing agents in the CNT dispersion and in the already existing thin water film. Thereby a self-filtering effect is avoided and thus assures a widely homogeneous thin self-entangled CNT film coating (Supplementary Fig. 2) well below 1 µm over several mm penetration into the 3D network (ensuring aspect ratios >10^4^). A more detailed description of the self-entangled layer formation is presented in the Supplementary Discussion together with a 2D model (Supplementary Fig. 3). By repeated infiltration after drying (which reduces the effect of the dispersion agent on the already deposited CNTs), the CNT concentration increases leading to the deposition of thicker CNTTs in the form of felted self-entangled networks (Fig. 1g–i). In the final stage, the drying process tends to transport CNTs in minimal surfaces pronounced at junctions in the ceramic network, which results in the formation of CNT nano-nets spanning between the legs of the tetrapods (Fig. 1e). The thickness of the self-entangled CNT network layers ranges approximately from 50 nm to 1000 nm (Fig. 1h, i), depending on the infiltration time. Samples with different CNT concentrations at places where arms of the tetrapods were broken were investigated (Fig. 1h, g) and the thickness of the CNT layer was measured, which varied only slightly throughout the complete 3D samples. The interwoven CNT networks formed can be compared to the so-called 2D buckypapers, which are sheets made of CNTs (Fig. 1g, i)^8,10^. In general such CNT sheets are formed via the deposition of suspended nanotubes onto a suitable 2D substrate. During drying, the CNTs form highly porous, interwoven networks, consisting of randomly arranged nanotubes, which are held together only by felting and van-der-Waals forces.

In contrast to reported synthesis processes in literature, the present strategy offers a high degree of fabrication diversity and is not limited to the material presented here. In fact, a large number of permutations and combinations (e.g., other 1D and 2D nanomaterials) can be adopted for realizing multifunctional 3D nanostructures. An example is presented in Supplementary Fig. 4, showing a highly porous 3D polymer structure produced by the same method.

**Fig. 2:**
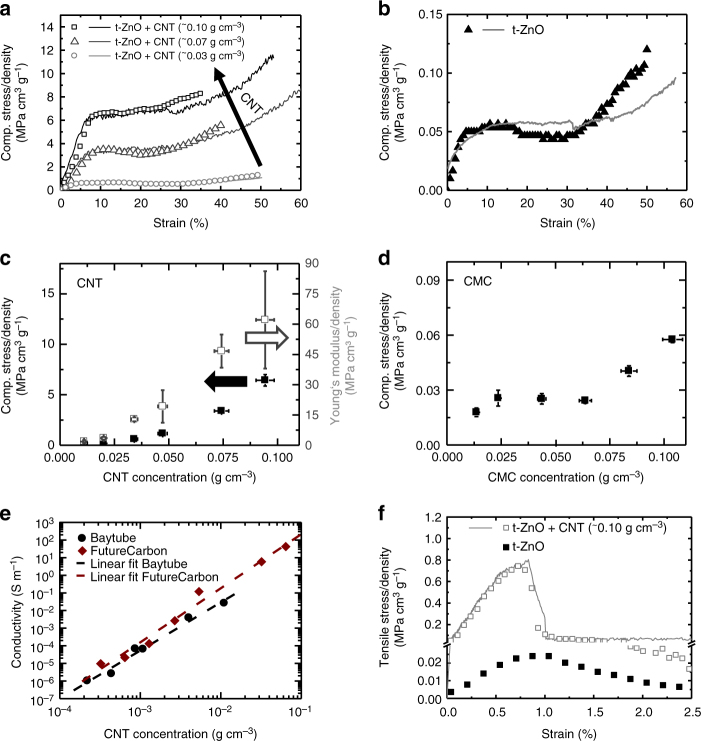
Mechanical and electrical properties of the CNTT/t-ZnO composite structure. **a** Compressive stress–strain curves for CNTT/t-ZnO networks containing different amounts of CNT normalized to the density of each structure (solid curves represents the corresponding results from FEM); **b** Compressive stress–strain curves for the pure t-ZnO network normalized to density (solid curve represents the corresponding FEM result); **c** Compressive strength and Young’s modulus (normalized with respect to density) vs. CNT concentration (error bars are s.d.); **d** Compressive strength (normalized with respect to density) vs. cellulose (CMC) concentration (stabilizing agent in the CNT dispersion) (error bars are s.d.); **e** Conductivity as a function of carbon nanotube concentration for two different CNT dispersions (CarboDis TN (Future Carbon), and Baytubes DW 55 CM (Bayer MaterialScience)); **f** Stress–strain curves under tensile loading for the pure ceramic network and for a network containing 0.1 g cm^−3^ carbon nanotubes (values are corrected by the density of the structures; solid curves represent the corresponding results from FEM). Please note that the template density was set to 0.3 g cm^−3^ for all mechanical and electrical measurements shown

The mechanical and electrical properties of the CNTT/t-ZnO composites are presented in Fig. 2, revealing a surprisingly high compressive strength increase by more than 2 orders of magnitude. Compressive strength has been enhanced by more than a factor of 147, i.e., from 0.017 MPa (equal to a specific strength of ~0.057 MPa cm^3^ g^−1^) of the reference ceramic network to almost ~2.5 MPa (equal to ~6.4 MPa cm^3^ g^−1^) for the maximum coated network, adding only 25 wt%. The porosity changes from 93 to 87% after infiltration, for the maximum CNT loading of 0.1 g cm^−3^. However, due to the addition of CNTs, pores in the nanometer range are introduced, next to the large pores (µm-range) of the 3D template. The resulting highly porous CNTT-ceramic 3D composite structure with a height of 6 mm is thus able to carry more than 10^5^ times of its own weight. Some typical stress–strain (compression) behaviors of CNTT/ceramic composites with varying CNT concentration, as well as for that of the pure ceramic (reference) are given in Fig. 2a, b respectively. In Fig. 2c, the maximum compressive strength and the Young’s modulus values are plotted as a function of CNT concentration in the composite for templates having a density of 0.3 g cm^−3^. Additional measurements and discussions regarding the influence of the template density on the mechanical properties of the CNTT/ceramic composites can be found in Supplementary Fig. 5. As depicted, the CNT concentration was varied between 0 and 100 mg cm^−3^. Furthermore, before testing all samples were dried overnight to avoid any water contaminations, which could lead to an improvement in the mechanical properties based on capillary forces between the individual particles (CNTs), as well as between the CNTs and the ZnO. Furthermore, we observed, that for templates having a very low density (<0.15 g cm^−3^) repeated infiltration leads to a shrinkage of the template, which might be related to the high capillary forces between the individual CNTs during drying, leading to high tensile forces and thus to the shrinkage of the template (similar to the effect as in the fibers of wet paper)^42^. Moreover, since the aqueous CNT dispersion was stabilized by carboxymethyl cellulose (CMC), measurements of the reference material without CNTs have also been carried out (Fig. 2d). The density of the utilized ceramic was kept constant at 0.3 g cm^−3^ for all the composite specimens, and at least three samples were measured for each data point shown. From the stress–strain curves it can be clearly seen that both the compressive strength and the Young’s modulus increase with an increasing amount of infiltrated CNTs (Supplementary Table 1). The reason for the drastic increase in compressive strength is mainly attributed to the structured architecture within the composite, converting the compressive stress on the ceramic into tensile strength of the individual CNTs inside the CNTTs. Thus a tensional integrity is formed (tensegrity), which is a structural principle in architecture, based on the use of isolated components in compression inside a net of continuous tension, allowing for a mechanical stable network under compressive stress, by redirecting the force into tension. In such a configuration, the structural collapse is only possible if the cables yield or the rods buckle^43^. The results obtained from FEM simulations closely confirm the enhancement in strength and stiffness with increasing CNT content in the hybrid network, directly related to the amount of infiltration time (Fig. 2c and Supplementary Fig. 6). Infiltration time is related to the thickness of the coating in this model (see Methods for details). Similar to the role of steel rebar in reinforced concrete, the CNT net with their superior mechanical tensile properties can also seen as a reinforcement of the ZnO tetrapods. Moreover, their contribution is maximized, being far from the cross-section centroid. Additionally, the structure of the CNTT/t-ZnO composite can be mimicked and compared to a traditional bamboo lashing^44,45^, as depicted in Fig. 1a. In general lashing is known as an arrangement of ropes or webbing to combine two or more items into one rigid structure. In case of traditional bamboo lashing, several flexible filaments are wound around the intersecting points of two bamboo sticks, leading to a lightweight framework with a very high structural strength. The same holds true for the here presented CNTT/t-ZnO structure, in which the interconnection points of the individual tetrapodal legs are combined by simple wrapping with webs made of self-entangled CNTs, as shown in Fig. 1d, e. As holds true for traditional lashing techniques, exerting a force onto this branched structure leads to an employment of the ropes/CNTs in tension, even though the specimen is compressed. This can be clearly observed in Supplementary Fig. 7, which shows that a ceramic network coated with CNTs exhibit a higher restoring force in comparison to the reference material. This indicates that the individual CNTs or networks were stretched during compression and are released after removal of the force (Supplementary Movie 2). Interestingly, the SEM investigations of mechanically compressed composites containing higher amounts of CNTs show a different fracture behavior in comparison to the reference networks. Behaving like a clamped cantilever^46^ and as per our observations, the pure ZnO tetrapods tend to break at the junctions between the central core and the legs, where the maximum stress arises. However, for CNT-coated t-ZnO networks, it could also be observed that individual legs get fractured (Supplementary Fig. 8), which is a clear indication that the entangled CNT networks formed lead to an overall reinforcement of the ceramic micro-architecture, especially at the interconnection points of the tetrapods within the network and thereby redirecting the stress. Furthermore, even if the structure is broken, it does not collapse due to the felted self-entangled CNT network around it (Supplementary Fig. 8). As indicated in Fig. 2a, c the reinforcing effect is rather small for low CNT concentrations. However, the stepwise addition of CNTs increases the self-entanglement and thereby the compressive strength value as well as the Young’s modulus (up to 2.5 MPa (normalized by density 6.4 MPa cm3 g^−1^) and 24.5 MPa (normalized by density 62 MPa cm^3^ g^−1^). From FEM simulations a strength of 16.3 MPa and a tensile modulus of 3.1 GPa was estimated for the formed CNT film. Composite specimens containing only CMC exhibit only a negligible increase in compressive strength.

The self-entangled CNTT structural network not only affects the compressive strength, but also increases the overall tensile response of the ceramic network by nearly two orders of magnitude. In Fig. 2f a direct comparison between a highly infiltrated and a non-coated network under tensile stress is presented. It can be clearly seen that the 3D composite structure exhibits a specific tensile strength of ~0.8 MPa cm^3^ g^−1^ (equal to ~0.3 MPa) whereas the uncoated highly porous ceramic has only a strength of around 0.02 MPa cm^3^ g^−1^ (it should be noted that measuring the tensile properties of such a fragile structures is quite complicated, several samples have been measured showing a tensile strength between 3 × 10^–3^ and 7 × 10^–3^ MPa; the highest value is shown here). Furthermore, the specific Young’s modulus increases from ~3 MPa cm^3^ g^−1^ for the reference template to ~112 MPa cm^3^ g^−1^ for the CNTT/t-ZnO composite, which is equal to a factor of ~40 (Supplementary Table 1). It should be also noted, that the strain at which the maximum stress is reached is rather low (~0.7%) when compared to compression (~7%). During tensile testing the self-entangled CNT networks become disentangled. The force that needs to be overcome is mainly attributed to van-der-Waals forces, as in the case of 2D buckypapers^9^. However, when those networks are infiltrated with a polymer, even a weak one like Silicone (e.g., PDMS), a 3D composite is formed. Even though no chemical matrix-filler binding exists, the external applied stress will be entirely transferred to the felted structure of the CNTs and thus increasing the mechanical properties at least by the same strength observed for the unfilled CNTTs.

For conductivity measurements 3D CNTT/t-ZnO composite networks with varying CNT concentration (between 0.2 and 10 mg cm^−3^ in the case of Baytubes DW 55 CM and 0.32–64 mg cm^−3^ in the case of CarboDis TN) were synthesized and detailed measurements were carried out (Fig. 2e). In both cases, the conductivity *σ* varies with the CNT concentration (*c*) approximately with a cubic law (*σ*∝*c* ^3^) and the values ranging from 1 × 10^–6^ to 2.6 × 10^–2^ S m^−1^ at CNT concentrations of 2 × 10^–4^ and 1.1 × 10^–2^ g cm^−3^, respectively. The approximated cubic law is in agreement with theoretical results obtained from statistical percolation theory^47^. The high conductivities at low CNT concentrations are directly related to the unique structural hierarchy of the network. The template, due to its tetrapodal shape, forms already a 3D percolation network. Therefore, only a few CNTs are needed to form a 3D percolation network on the pre-existing network, compared to the case in which the CNTs need to be arranged in the complete volume. Additionally, only a few dead ends (unconnected tetrapodal arms) exist and thus nearly all the surface of the template contributes to the conductivity. This fact changes drastically in the case of other porous structures having many parallel conductive pathways, which do not contribute to the overall conductivity. The pronounced increase in conductivity with increasing CNT concentration is mainly attributed to the increase in the number of interconnection points, leading to the formation of new conductive pathways. At very low CNT concentrations, no ohmic behavior could be observed, indicating that at such concentrations (<2 × 10^–4^ g cm^−3^) no interconnected conductive pathways exist in the complete 3D sample.

### 3D self-entangled CNTT networks

**Fig. 3:**
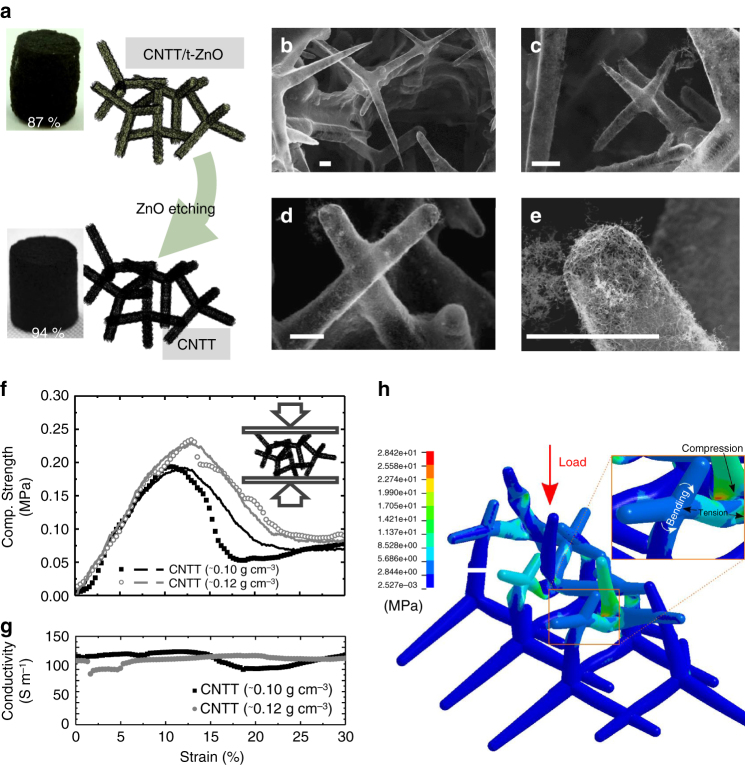
Overview of the 3D CNTT structures (0.12 g cm^−3^) obtained from CNTT/t-ZnO composites and their electrical and mechanical characteristics. **a** Schematic representation of the fabrication process, showing the CNTT/t-ZnO structure before and after etching with HCl (1 M in water); **b**–**e** SEM images of the resultant 3D CNTT assembly at different magnifications showing a hollow interconnected tubular structure consisting of self-entangled carbon nanotubes (scale bar: 5 µm); **f** Compressive stress–strain curves for 3D CNTT assemblies showing a high structural strength of up to 0.24 MPa (dots represent experimental measurements, continuous line FEM simulation); **g** Conductivity (~130 S m^−1^) during compression test; **h** FEM image showing the tensile behavior of the CNTT arms under a global compressive load, indicating that the CNTs are employed in their tensile properties, even though the structure is compressed

The highly porous 3D CNTT assembly is obtained just by removing the ceramic template of the CNTT/t-ZnO composite using a simple HCl etching process followed by subsequent critical point drying (Fig. 3a). The resultant architecture consists of hollow tube-shaped self-entangled CNT networks (Fig. 3b–e). Thereby, only samples containing at least 0.07 g cm^−3^ were stable after the drying process, showing a ghost-like 3D assembly of CNTs with very thin tube walls (Supplementary Fig. 9). The treatment of CNTs using HCl is a well-established method for the removal of impurities and causes no damage to the CNTs under mild conditions (1 M HCl was used in this study)^48^. In Fig. 3f, stress–strain curves for 3D CNTT structures having a CNT concentration of 0.10 g cm^−3^ and 0.12 g cm^−3^ are depicted together with results of numerical simulation^49^. The corresponding conductivity curves for the two samples are presented in Fig. 3g. The conductivity has been nearly constant during uniaxial compression with values ~130 Sm^−1^. The stress–strain curve shows a linear elastic regime for strains <13%. After that, the ultimate compressive strength is reached with a value of ~0.24 MPa. The highly porous structure (94%) with a length of 6 mm is thus able to bear more than 40,000 times of its own weight. A lower CNT concentration leads to a less stable network (Supplementary Fig. 9), due to less self-entanglement. Compared to the CNTT/t-ZnO composite networks, this value is only one order of magnitude lower, demonstrating that the pure 3D CNTT assembly still exhibits excellent mechanical properties and is able to bear 2.4 kg cm^−2^, even having a high porosity of ~94%. As mentioned beforehand, the high strength of the network is directly related to the double-hierarchical architecture. FEM simulations shown in Fig. 3h (see also Supplementary Movie 3) reveal that under a global compression scenario the CNTT film shows regions in which tensile forces act on the coating, confirming again that the CNTs are employed in their extraordinary tensile properties. However, after compression the structure is not able to recover its original shape, both due to damage of the CNTT (rearrangements in the CNTs network) and to the mutual slip of the tetrapods, strongly increasing the toughness modulus (area under the stress-strain curve). This is also in agreement with the already existing literature reports about other types of 3D CNT assemblies prepared by wet chemical approaches and with aerographite tetrapod networks^11,12^.

### Stretchable 3D CNTT/polymer composites

**Fig. 4:**
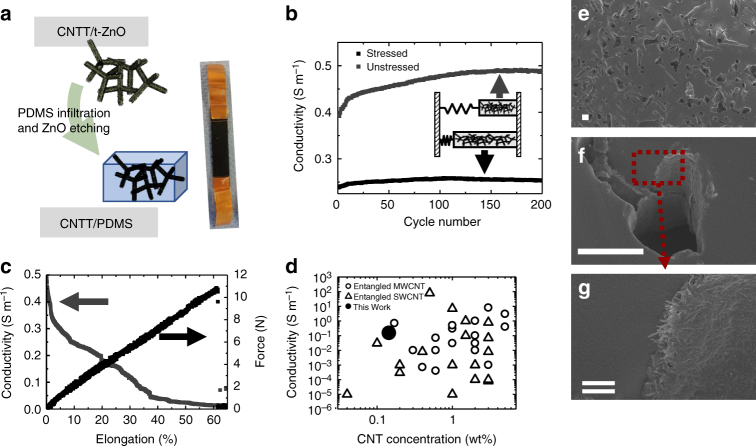
Overview of the CNTT/PDMS porous stretchable conductors and their mechanical and electrical characteristics. **a** Left: schematic representation showing the PDMS infiltration into the CNTT/t-ZnO composite and subsequent ZnO etching with HCl. Right: stretchable porous conductor used for conductivity measurements; **b** Conductivity against cycle number for the unstressed (0% elongation) and the stressed (13.3% elongation) state, showing an increase in conductivity for both states during cycling (CNT concentration was 11.3×10^–3^ g cm^−3^); **c** Mechanical and electrical properties under tensile stress of a porous stretchable conductor having a CNT concentration of 11.3×10^–3^ g cm^−3^. With increasing elongation, the conductivity decreases. **d** Comparative log–log plot of maximum conductivity as a function of CNT weight fraction (adopted from ref. ^47^); **e**–**g** SEM images of the CNTT/PDMS composites showing the interconnected channels with CNT decorated walls after template removal by HCl (Scale bars: single line: 10 µm, double line: 1 µm)

Moreover, the availability of such highly porous networks offers the possibility of fabricating new varieties of CNTT based 3D composites. An exemplary case of 3D CNTT/polymer architectures using PDMS is demonstrated here, showing a unique self-enhancing effect in conductivity during cycling based on the double-hierarchical architecture. The highly conductive and mechanically stable CNTT/t-ZnO composites can be easily infiltrated with appropriate polymers and the ZnO template can be removed if needed, thereby enabling the fabrication of stretchable porous conductors in form of interconnected channels coated with CNTs (Fig. 4a). The channels produced by wet chemical template removal (HCl) are clearly visible, having a layer of felted self-entangled CNTs at their walls (Fig. 4e–g). According to literature no damage to the used elastomer is caused by the etching process^50,51^. The porosity of the resultant CNTT/polymer composite is in the order of ~7% after etching of the highly porous ceramic template. Due to the low amount of CNTs used for the CNTT/polymer composites, their volume is negligible. In general the electrical conductivity of CNTT/polymer composites depends on several factors, including the type of CNTs (single, double, or multi-walled), as well as on their length, diameter, synthesis method, etc.^47,52^. However, the characteristics of the CNTs used seem to be less important than those of the polymer matrix used and the dispersion methods required to obtain a homogeneous CNT distribution and thereby a low percolation threshold. The larger van-der-Waals interactions between individual CNTs compared to those between polymer-polymer chains often lead to the aggregation and thereby leading to an increase in the percolation threshold^47,52,53^.

### Self-enhancing effect in PDMS/CNTT composites

The here shown anisotropic distribution of entangled multi-walled CNTs in the CNTTs can be used to decrease the percolation threshold for all types of castable polymers as was already discussed with respect to the electrical properties of the CNTT/t-ZnO composites, but also another unique mechanism can be observed, such as a self-enhancing effect in conductivity during cyclic stretching. Figure 4c shows the force–strain curve as well as the conductivity until failure of a CNTT/PDMS composite sample. From the graph it can be clearly seen that the conductivity is decreased with increasing elongation of the sample, having an initial conductivity of ~0.52 S m^−1^. The decrease in conductivity is rather pronounced in the first 3% of elongation, with a conductivity drop of up to ~0.35 S m^−1^. After that the conductivity decreases more slowly up to the breaking point (at ~60% strain). This behavior can be attributed to the stretching of the felted CNTs. Thereby some interconnection points between the individual CNTs are broken, which leads to an overall decrease in electrical pathways and thus to a decrease in conductivity (Supplementary Fig. 10). This behavior is well known for stretchable conductors^54^. A rather unexpected phenomen is the increase in conductivity caused by cycling, similar to the work hardening in mechanics. An example is shown in Fig. 4b for a PDMS/CNTT composite having a CNT concentration of 11.3 × 10^–3^ g cm^−3^. It can be directly observed, that the stressed state always shows a lower conductivity compared to the unstressed one, having an initial spread of ~0.15 S m^−1^. However, when cycling the samples at small elongations (~13%), a self-enhancing effect in conductivity can be observed over time. This effect is higher for the unstressed state, showing an increase in conductivity from ~0.39 S m^−1^ to ~0.48 S m^−1^ after 150 cycles, meaning that the conductivity increases by almost 23%. After that, the conductivity stays rather constant. The effect can be also seen in the stressed state, although, it is rather small compared to the unstressed one. The same behavior can be also observed in samples having a lower CNT concentration of 1.8 mg cm^−3^ (Supplementary Fig. 11), showing an initial conductivity of around 0.136 S m^−1^ which is increased to ~ 0.15 S m^−1^ after 150 cycles.

The self-enhancing effect observed is rather unexpected and has not been reported earlier for other stretchable conductors^29,55–58^ to the best of our literature knowledge. On the contrary, most of the stretchable conductors suffer from a decrease in conductivity after several elongation cycles^29^. For the observed increase in conductivity, we propose the following mechanism as illustrated in Supplementary Fig. 10: in the initial state, there will be only a few conductive pathways through the CNT network. By stretching one of the hollow tubes (with the CNTs at the walls) in the direction of its length, some of those conductive pathways will be destroyed, leading to a decrease in conductivity during stretching. However, during the stretching, some CNTs will become orientated and aligned along the direction of the applied force. This effect was also observed in other publications^59,60^. Due to the alignment the interface area between individual CNTs enlarges, leading to higher van-der-Waals forces between the individual CNTs. If the force is released again, the elastomer (PDMS) will retain its original shape, however, due to the higher van-der-Waals forces and alignment between the individual CNTs, some of the CNTs will stay orientated and will not retain their original shape/position. Thereby, new conductive pathways are formed, leading to an increase in the overall conductivity. The more often the sample is stretched, the more CNTs are aligned and the new electric pathways are formed leading to an increase in the overall conductivity. At some point, most of the CNTs are oriented and no further increase in conductivity can be observed upon further cycling. Furthermore, due to the 3D arrangement of pores in the PDMS caused by the tetrapodal template, this effect will only influence some of the CNTs, mainly the ones at the tubes that are oriented in the direction of the applied force. Thus, due to the double-hierarchical structure a unique self-enhancing effect in conductivity can be observed.

### Low percolation threshold

In addition, the double-hierarchical structure of the CNTT composite is also a very decisive factor for the observed high conductivities even at low CNT loadings. In Fig. 4d, several literature values for entangled multi-walled CNTs as well as entangled single-walled CNTs in an epoxy matrix are shown (adopted from ref. ^47^) and compared to present composites. For the calculations, the density of the multi-walled CNT used was estimated to be 1.4 g cm^−3^. In contrast to all other systems using entangled multi-walled CNTs, the preparation strategy presented here allows for the simple fabrication of highly conductive stretchable conductors, using extremely low CNT concentrations (1.8 mg cm^−3^). It is very important to mention here, that this remarkably high value of conductivity could be achieved, even using the commercially available CNT dispersion. The high conductivity at such low CNT concentrations can be directly correlated to the structure and the high porosity of the ceramic template. Infiltrating the templates with CNTs results in the formation of conductive pathways only on the surface of the templates. Thus, the volume in which the CNTs need to be distributed to form a percolated network is strongly decreased. This simple strategy enables the formation of conductive pathways even at very low CNT concentrations, reducing the percolation threshold, without the need of complicated mixing and dispersing required by other processes.

## Discussion

In summary, a simple strategy for the versatile fabrication of macroscopic highly porous 3D architectures consisting of interconnected tubes made from self-entangled CNTs (CNTT) has been demonstrated. This self-organized process not only allows for the formation of CNTT networks, but also the formation of 3D nano-architectures from almost any wet chemically dispersed desired nanoscale structure (1D, 2D, etc.). In fact, several different kinds of low-D nanomaterials can be combined in a tube-shaped 3D structure, enabling the fabrication of multifunctional inorganic-carbon-polymer hybrid 3D nano- and microarchitectured materials for advanced applications. Additionally, the ceramic network can be easily removed by simple chemical etching, which offers a lot of nanostructuring opportunities in the direction of new interesting highly porous 3D composites without the need for high temperatures or vacuum equipment. For example: CNTT-ceramic composites, 3D CNTT stand-alone nano-architectures, CNTT-ceramic-polymers, as well as CNTT-polymer composites have been demonstrated. By the formation of self-entangled CNT networks around the highly porous 3D ceramic a nanostructure with mechanical properties based on the principle of tensegrity was introduced. The specific mechanical and electrical properties of the highly ceramic network were enhanced by several orders of magnitude, forming an efficient combination of two material classes. Their complex shaped architecture together with their properties makes them a versatile base and promising candidate for applications in the field of sensors, nanoelectronics, and especially metal-free and functional filters.

Moreover, due to its unique double-hierarchical tube-like structure and the high density of CNTs, the stand-alone CNTT structure shows a higher mechanical stability compared to other 3D CNT assemblies reported before that are based on wet chemistry (Supplementary Fig. 12) and can be easily functionalized with other low-D nanomaterials with a lot of potential application, e.g., as an electrode for Li–S batteries or tissue engineering. Furthermore, embedding these 3D buckypaper-like structures into a polymer allows for an improvement in the mechanical properties, even though no chemical matrix-filler binding exists. Finally, the CNTT-polymer composites (e.g., infiltrated with PDMS) can contribute with their remarkable flexibility, stretchability and fatigue resistant properties to the realization of a new generation of electronic devices.

## Methods

### Fabrication of CNTT/t-ZnO composites, 3D CNTT assemblies, and PDMS/CNTT composites

The t-ZnO ceramic networks used for composite manufacturing were produced by a simple flame transport synthesis^33^. Zinc powder with a grain size of 1–10 µm was mixed with polyvinyl butyral in a mass ratio of 1:2. The mixture was then heated in a muffle furnace with a heating rate of 60 °C min^−1^ up to 900 °C for 30 min. After that a loose powder of typical ZnO tetrapods was obtained, which was pressed into pellets (height = 6 mm, diameter = 6 mm), having a density of 0.3 g cm^−3^. Reheating the pellets for 5 h at 1150 °C leads to the formation of junctions between the tetrapods. For CNTT/t-ZnO composite preparation, commercial aqueous CNT dispersion (Baytubes DW 55 CM, Bayer MaterialScience) was diluted with water to a CNT concentration of 1% wt. Additional experiments were carried out using other MWCNT dispersions, namely CarboByk 9810 (BYK-Chemie) and CarboDis TN (Future Carbon). To reduce the amount of agglomerates, the dispersion was treated with ultrasonication for 20 min before each infiltration step. While being exposed to 40 °C the t-ZnO pellets were infiltrated with the aqueous CNT dispersion using a self-engineered computer-controlled syringe and subsequently dried for at least 1 h. In this case, infiltration means that the templates were completely filled with dispersion, until no more dispersion was taken up by the 3D network. By repeating this process several times the CNT concentration can be adjusted. For the preparation of the 3D CNTT assemblies, the previously prepared CNTT/t-ZnO composites were placed in a 1 M HCl for at least 4 h to remove the template. After that, the samples were washed with deionized water, which was replaced by pure ethanol (5 times washing), which is necessary for the subsequent critical-point-drying process using an EMS 3000.

Preparation of the stretchable porous conductors was carried out by infiltrating CNTT/t-ZnO films (60 × 10 × 0.5 mm) with PDMS (Sylgard 184). A pure PDMS backbone of 0.5 mm height was added for stability purpose. To get rid of any air inclusions after infiltration, the samples were degassed in an exsiccator for at least 1 h and afterwards cured under ambient conditions at 80 °C in an oven. For the ZnO removal the samples were placed in a 1 M HCl bath for at least 5 days. The etched samples were dried in a vacuum oven for at least 2 days.

### Characterization

The morphologies and structure of the different structures were investigated by SEM (Zeiss Supra 55VP). For the measurement of the CNT layer thickness, at least 3 samples were investigated and the layer thickness was measured at the connection of broken tetrapod arms (a result from the SEM preparation). With respect to the electrical measurements, CNTT/t-ZnO composites as well as the pure 3D CNTT assemblies were connected from both sides to copper foil using silver paste to avoid high contact resistance. In analogy to that, films used to make PDMS/CNTT composites were connected with copper foil at both ends prior to PDMS prepolymer infiltration. Electrical properties were measured using as Keithley 2400 in 4-wire sense mode. The mechanical characterization of the CNTT/t-ZnO composites was carried out using a tensile-test machine (Zwick 1445) due to its high mechanical stability, which did not allow for simultaneous electrical measurements. Uncoated ZnO networks, CNTT assemblies, and PDMS/CNTT composites were characterized using a self-engineered micromanipulator set-up. In the case of tensile test, all samples were carefully glued between two metal plates using superglue (please note: only a thin layer of superglue was used) and a short H_2_ treatment was used to increase the viscosity of the glue to ensure a minimal sample penetration. Furthermore, all samples were dried overnight to avoid any water contaminations, which could influence both the electrical as well as the mechanical properties as discussed in the manuscript.

### Computational modeling

The geometry of tetrapodal elements of pristine ZnO networks was modeled with arms of hexagonal section with mutual dihedral angles according to the geometry of a regular tetrahedron. According to the SEM image of Fig. 3d the arm length *l* was assumed of 27 µm (tetrapod center to arm tip distance). The cross-section at the tetrapod joint is inscribed in a circle of diameter *d*_1_ = 5 µm while of *d*_2_ = 3 µm at the arm end, yielding to a tapered arm geometry (Fig. 3c, d). The spatial density of the tetrapods in the simulated volume (initial cell dimensions, prior to compression, is 100 × 100 × 100 µm^3^ for both ZnO-CNT and CNTT networks) is derived according to the density of the experimental samples (0.3 g cm^−3^ for the ZnO template). ZnO tetrapods were modeled with under-integrated solid element (spurious modes controlled). The CNT nets covering the surface of the ceramic network (and thus the CNTTs) are described as an equivalent continuum made of thick shell elements with selective reduced integration^61^, covering the ZnO tetrapods with nominal geometries. The thickness *t* of the CNT film after infiltration of the ZnO tetrapod networks was calculated—from the known concentration *c* of CNTs in the solution and the porosity of the ZnO template (Supplementary Table 1)—assuming a uniform coverage. The estimated thicknesses were of 0.46, 1.01, and 1.28 µm for the three analyzed ZnO-CNTs composites for the different CNT solutions as well as of 1.36 µm and 1.63 µm for the two tested CNTT networks, respectively. Thus, it is possible to derive a relation in the form *t* = *k*·*c* between the CNT thickness *t* and the CNT concentration *c* in the solution, where *k* = 1.136×10^−3^ cm^4^ g^−1^ from our estimate. These calculations result in density increments after infiltration all close to the experimentally observed values. The constitutive behavior of both phases (the ceramic network and the CNTs net) are modeled as linear elastic isotropic. Failure of the CNT film is allowed by an erosion algorithm, being imposed both a limiting stress and strain. In all simulations the compression load on the cell is applied via the moving rigid walls in displacement control which confines the tetrapod networks; the stress is determined from the total contact force at the rigid wall-tetrapods interface normalized by the sample network area, i.e., 100 × 100 µm^2^. Contact is implemented between the different tetrapods, and also self-contact is allowed for walls of the same CNTT tetrapod, assuming static and dynamic coefficients of friction of 0.2 and 0.1, respectively. Entanglement of tetrapods in CNTT networks was obtained by intersecting adjacent tetrapod arms, and welding superimposed tube nodes, at a distance equal to 0.4 *l* from the tetrapod arm end (see Fig. 3d for real entanglement geometry and Fig. 3h for the FEM model of entangled tetrapods).

### Data availability

The data that support the findings of this study are available from the corresponding authors upon request.

## Electronic supplementary material

Supplementary Information

Description of Additional Supplementary Files

Supplementary Movie 1

Supplementary Movie 2

Supplementary Movie 3

Supplementary Movie 4
