# Publisher Correction: Hierarchical self-entangled carbon nanotube tube networks

**DOI:** 10.1038/s41467-017-02372-9

**Published:** 2018-01-09

**Authors:** Fabian Schütt, Stefano Signetti, Helge Krüger, Sarah Röder, Daria Smazna, Sören Kaps, Stanislav N. Gorb, Yogendra Kumar Mishra, Nicola M. Pugno, Rainer Adelung

**Affiliations:** 1 Functional Nanomaterials, Institute for Materials Science, Kiel University, Kaiserstr. 2, 24143 Kiel, Germany; 2 Laboratory of Bio-Inspired and Graphene Nanomechanics, Department of Civil, Environmental and Mechanical Engineering, University of Trento, via Mesiano 77, I-38123 Trento, Italy; 3 Functional Morphology and Biomechanics, Zoological Institute, Kiel University, Am Botanischen Garten 1-9, 24098 Kiel, Germany; 4 Ket Lab, Edoardo Amaldi Foundation, Italian Space Agency, Via del Politecnico snc, 00133 Rome, Italy; 5 School of Engineering and Materials Science, Queen Mary University of London, Mile End Road, E1 4NS London, UK

Correction to: *Nature Communications* 10.1038/s41467-017-01324-7, Article published online 31 October 2017

The original version of this Article was missing the ORCID ID of Professor Nicola Pugno.

Also in the original version of this Article, the third to last sentence of the fourth paragraph of the Results incorrectly read “However, the stepwise addition of CNTs increases the self-entanglement and thereby the compressive strength value as well as the Young’s modulus (up to 2.5 MPa (normalized by density 6.4) and 24.5 MPa (normalized by density 62 MPa cm^3^ g^−1^).” The correct version adds the units “MPa cm^3^ g^−1^” to “6.4”.

Finally, in the original version of this Article, the *y*-axis label of Fig. 3f incorrectly read “Comp. strengthy”. The new version corrects that to “Comp. Strength”.

These errors have now been corrected in both the PDF and the HTML versions of the Article.

