# Supplementary material for: Hierarchical self-entangled carbon nanotube tube networks
Source: Nat Commun. 2017 Oct 31;8:1215. doi: 10.1038/s41467-017-01324-7 (PMC5662747; doi:10.1038/s41467-017-01324-7)
Supplement: Supplementary file 1 — Supplementary Information [file 41467_2017_1324_MOESM1_ESM.pdf]

Supplementary Figures

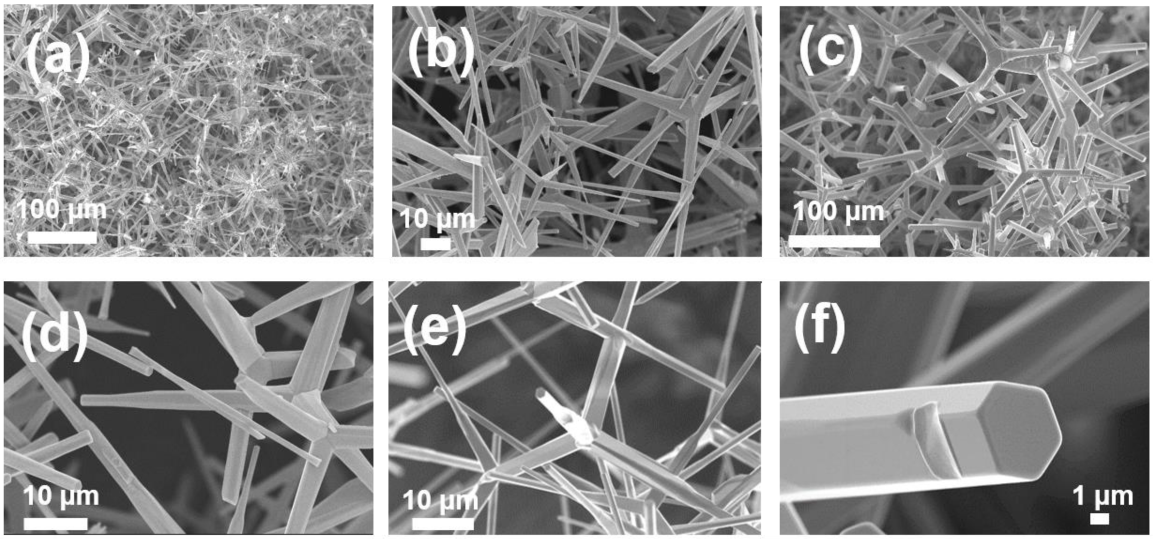

Supplementary Figure 1. Overview of the t-ZnO template. a,b,c,d) SEM images of the highly porous 3D template consisting of interconnected tetrapodal shaped ZnO particles, fabricated by a simple flame-transport synthesis with a mean diameter of around 25 µm and arm thicknesses of only a few micrometers; **e)** Junctions between the particles formed during sintering; **f)** High magnification SEM of a single tetrapod arm

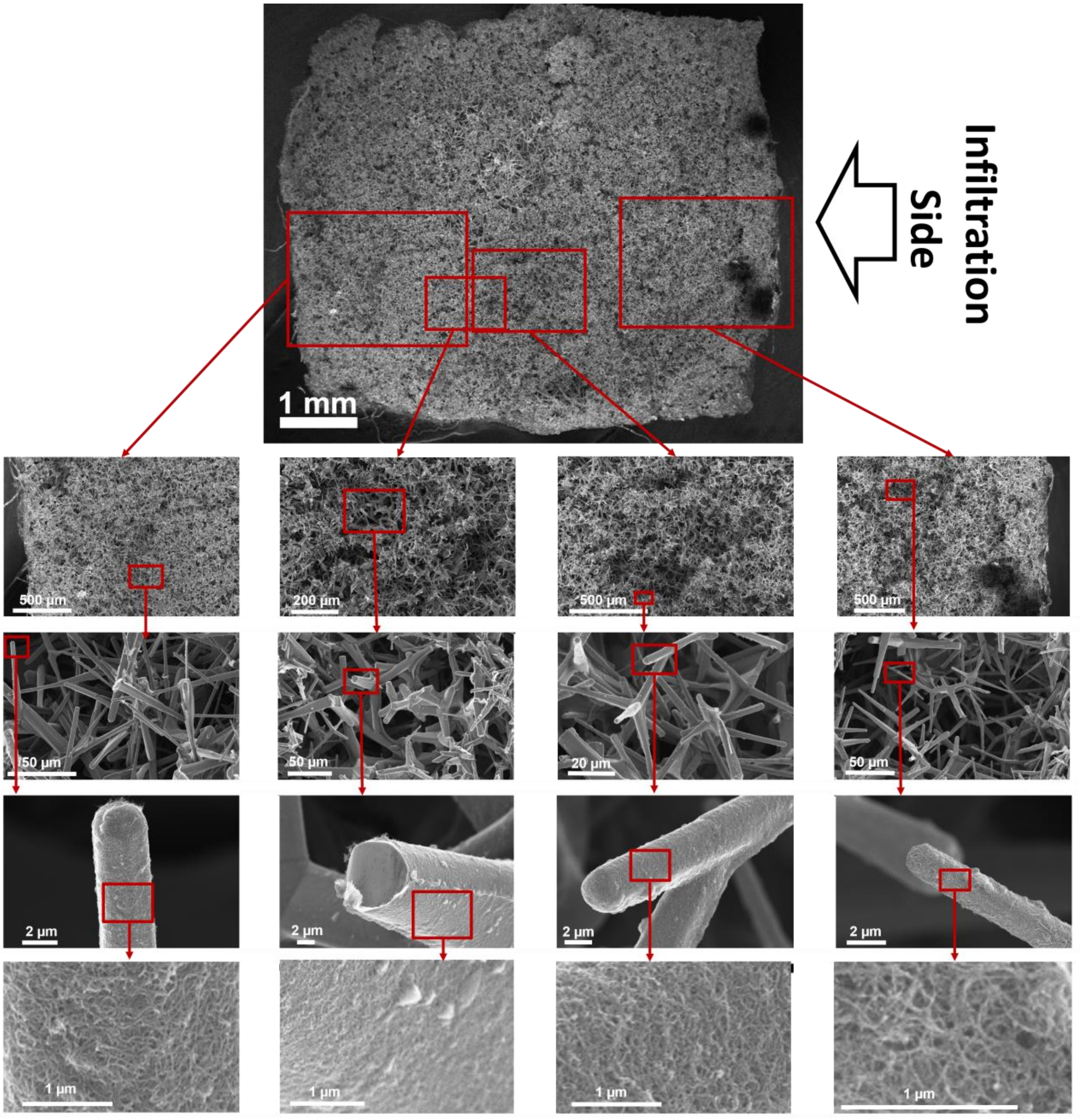

Supplementary Figure 2. Crossectional SEM investigation over a complete CNTT/t-ZnO 3D structure at different magnifications: The sample (height: 6mm, radius: 6mm) was infiltrated 12 times using a 0.5 wt% CNT dispersion (*CarboDis TN*). For the investigation, the sample was cut in the middle using a razor blade. The different areas indicate, that the coating of the template using the infiltration process is homogenous, even in the middle of the template (no self-filtering effect). Please note, that by cutting the sample, parts of the network get broken (e.g. detached CNT layers, broken tetrapod arms)

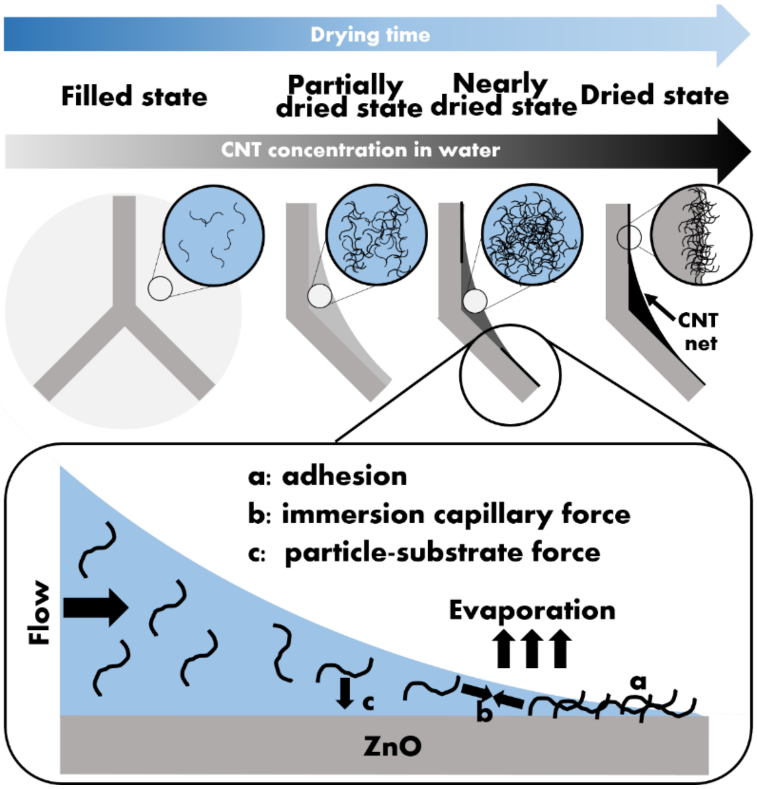

Supplementary Figure 3. Schematic illustration of the CNTT coating process. After infiltration with the CNT dispersion, the network is in the filled state (all pores are filled with CNT dispersion). As a result of the high porosity and the large pore sizes of the template the CNT concentration is the same all over the 3D structure. Due to the polarized surface of the ZnO some CNTs get directly attached (particle-substrate force) to it. Upon water evaporation, several menisci are formed between the interconnection points of the template. Due to evaporation losses, an outward fluid flow carries the dispersed particles to the pinned contact line of the meniscus. In this region, the particles start to form a homogenous layer due to immersion capillary forces and particle-substrate force (similar to convective self-assembly). This ensures a widely homogenous coating all over the 3D template structure. On top of that, spanning CNT nets between the interconnection points can be formed because of an increase in CNT concentration in the remaining water during drying.

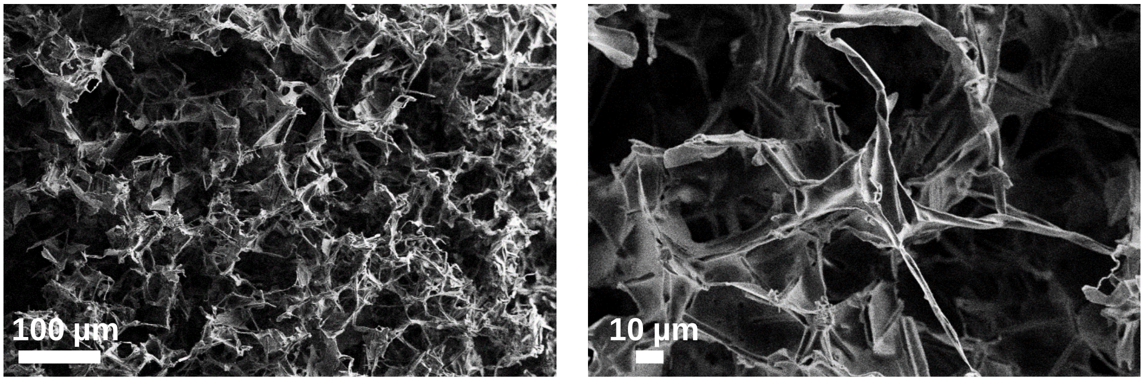

Supplementary Figure 4. Porous 3D interconnected polymer (PVDF) network after template removal.

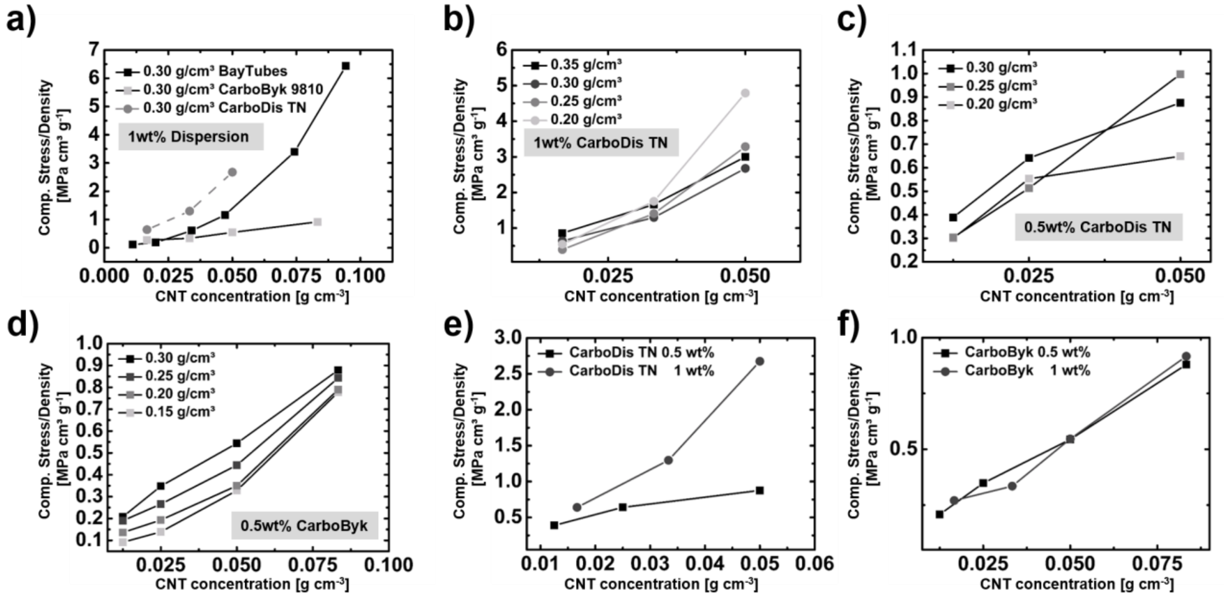

Supplementary Figure 5. Compressive Stress (normalized by density) vs. CNT concentration for different densities of the ZnO template for different MWCNT dispersions: a) Comparative plot between different MWCNT dispersions (CarboByk 9810 *CarboDis TN, Baytubes* DW 55 CM). The graph shown for the *Baytubes* is the same as shown in Figure 2c of the manuscript; **b)** CNTT reinforcement effect vs. CNT concentration with respect to the density of the template when a 1wt% CarboDis TN dispersion was used for the infiltration; **c)** CNTT reinforcement effect vs. CNT concentration with respect to the density of the template when a 0.5wt% CarboDis TN dispersion was used for the infiltration; **d)** CNTT reinforcement effect vs. CNT concentration with respect to the density of the template when a 0.5wt% CarboByk 9810 dispersion was used for the infiltration; **e)** CNTT reinforcement effect vs. CNT concentration for different CarbosDis TN concentrations in the dispersion used for the infiltration (template density 0.3 g cm^{-3}); **f)** CNTT reinforcement effect vs. CNT concentration for different CarboByk 9810 concentrations in the dispersion used for the infiltration (template density 0.3 g cm^{-3}).

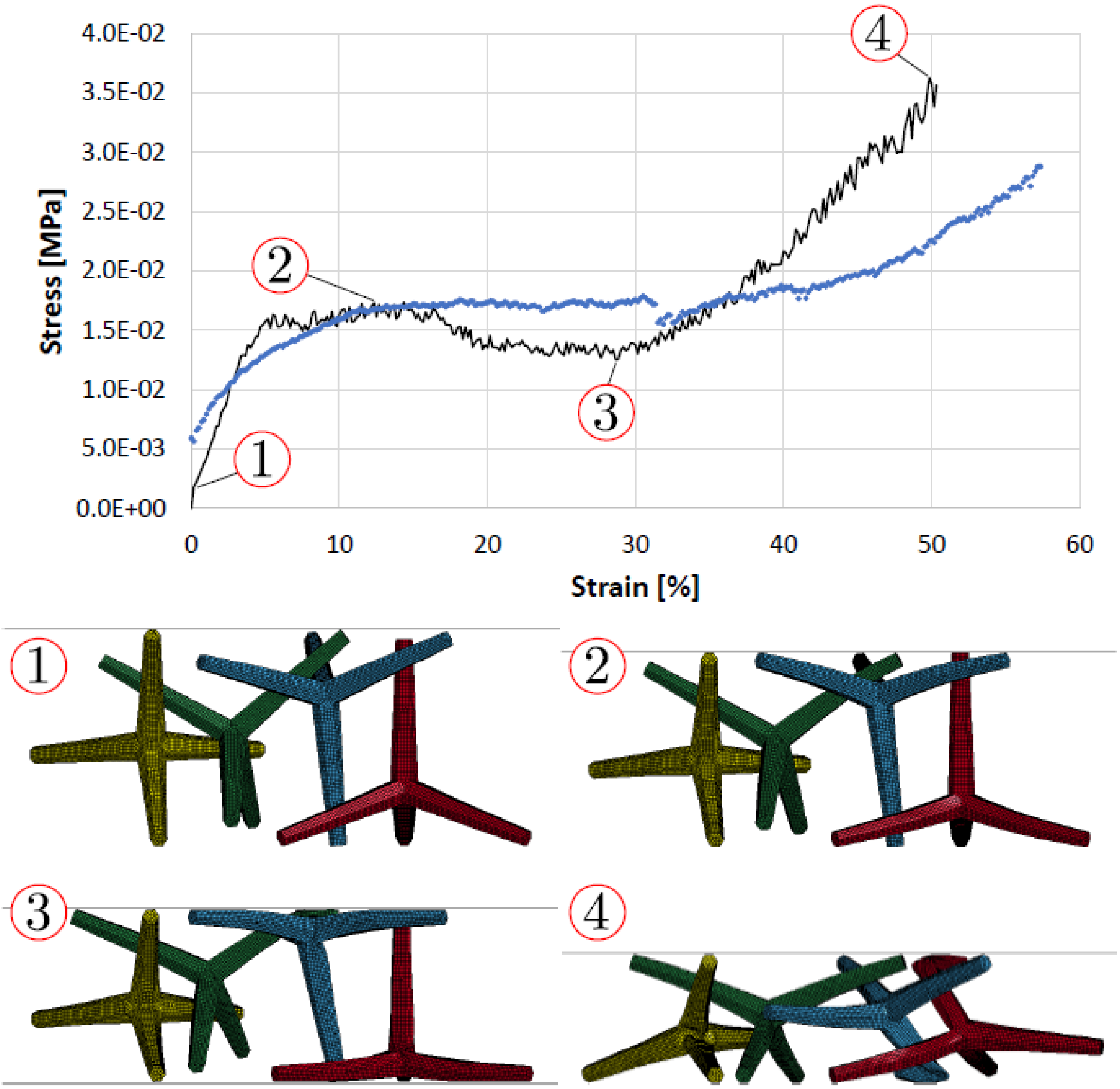

Supplementary Figure 6. Detail of compression experiments on t-ZnO network (see Figure 2). Blue dots represents experimental data while the black line is the result from FEM simulations. Snapshot of simulation at 4 different deformation levels are shown.

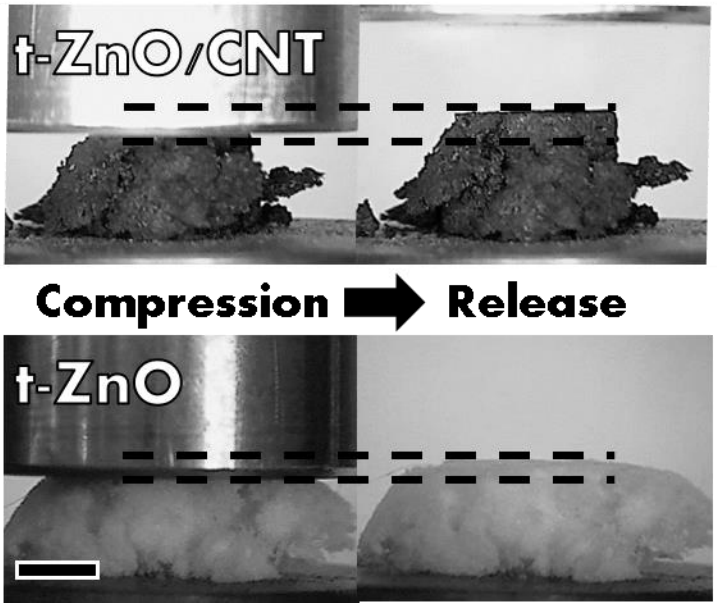

Supplementary Figure 7. Comparison between the pure template and a template containing 0.1 g cm^{-3} carbon nanotubes after compression showing that the carbon nanotube coating leads to a higher restoring force (scale bar: 3 mm). The whole compression experiment is shown in Video S2.

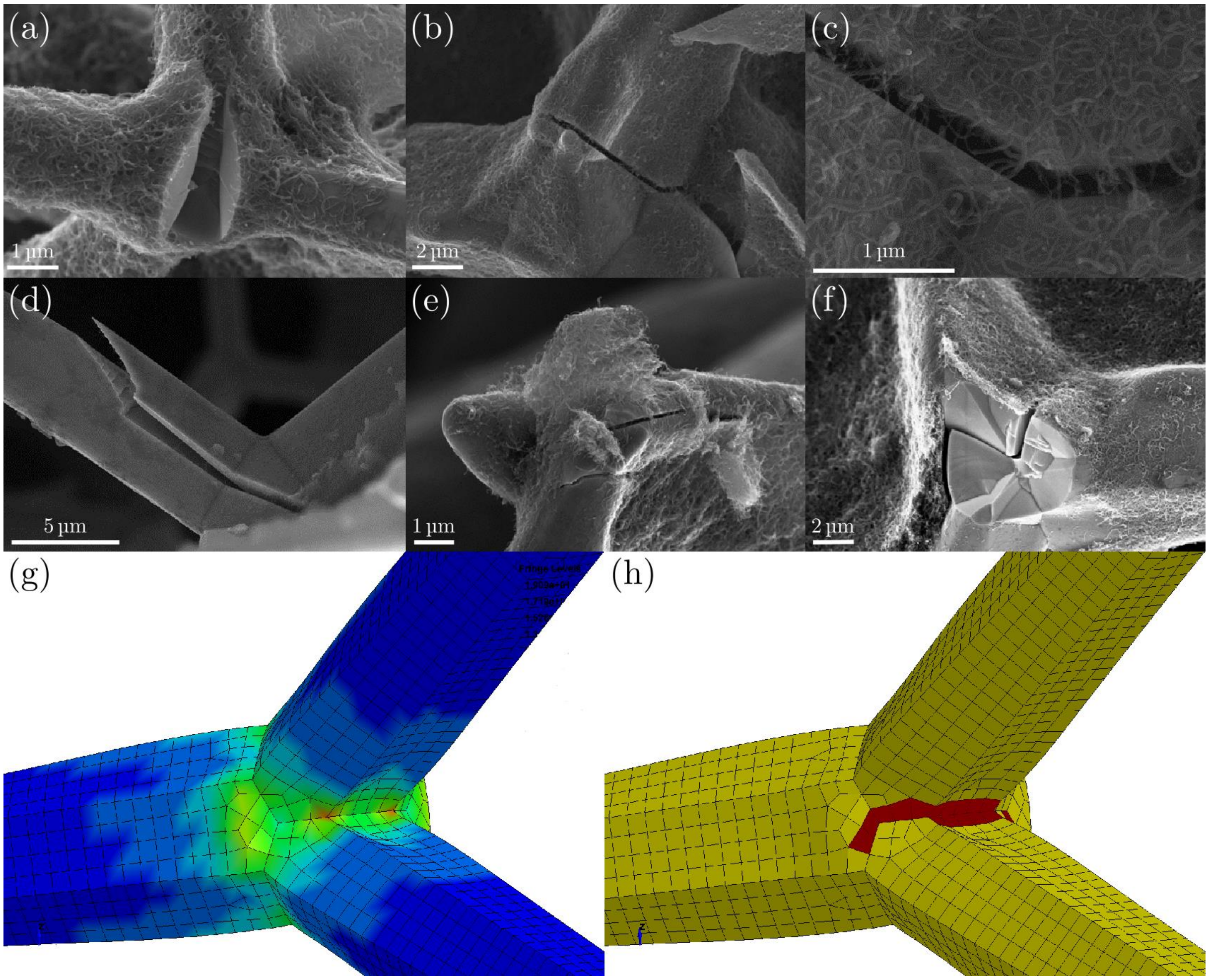

Supplementary Figure 8. Fractures sites of a t-ZnO/CNTT composite after compression: a-b) Fractures between the core and a leg of a tetrapod, which are still hold together by the carbon nanotubes; **c)** enlarged fracture site from b; **d-e)** Fractures inside the leg of a tetrapod; **f)** Fracture at the core of a tetrapod. **g-h)** Detail of FEM simulation showing the stress distribution at the t-ZnO/CNTT tetrapod joint which causes CNNT film failure. Elements of the film (in yellow) are eroded due to reach of the failure limit letting the t-ZnO below (in red) to be visible.

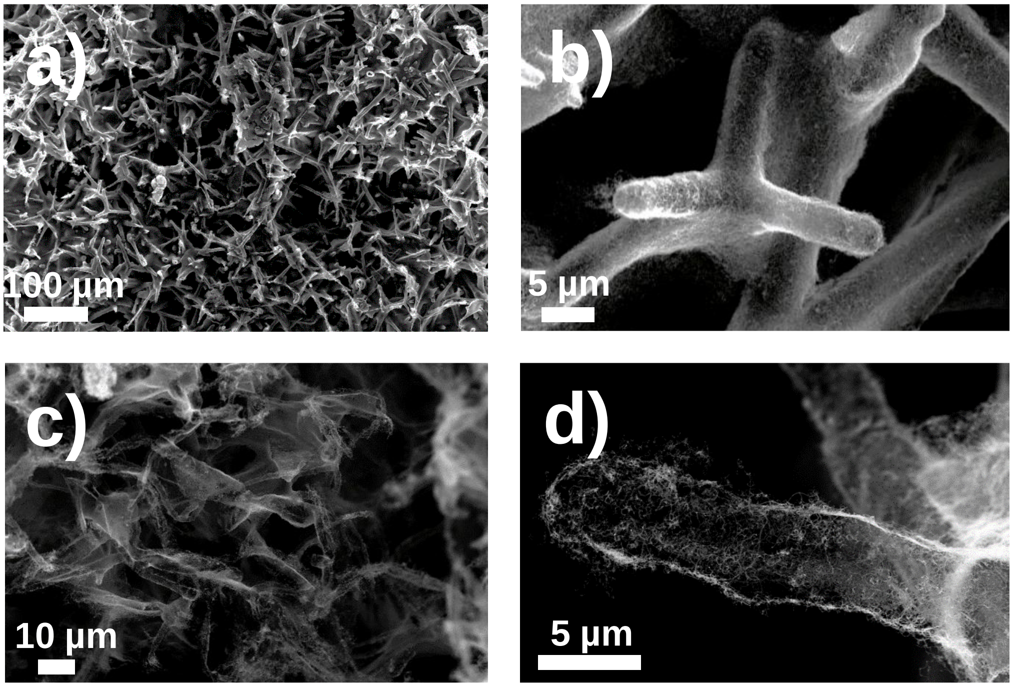

Supplementary Figure 9. 3D CNTT assembly at different magnifications: a-b) CNTT network (100 mg cm^{-3}) at low magnifications, showing the interconnected tubes; c-d) CNTT assembly having a density of 70 mg cm^{-3}.

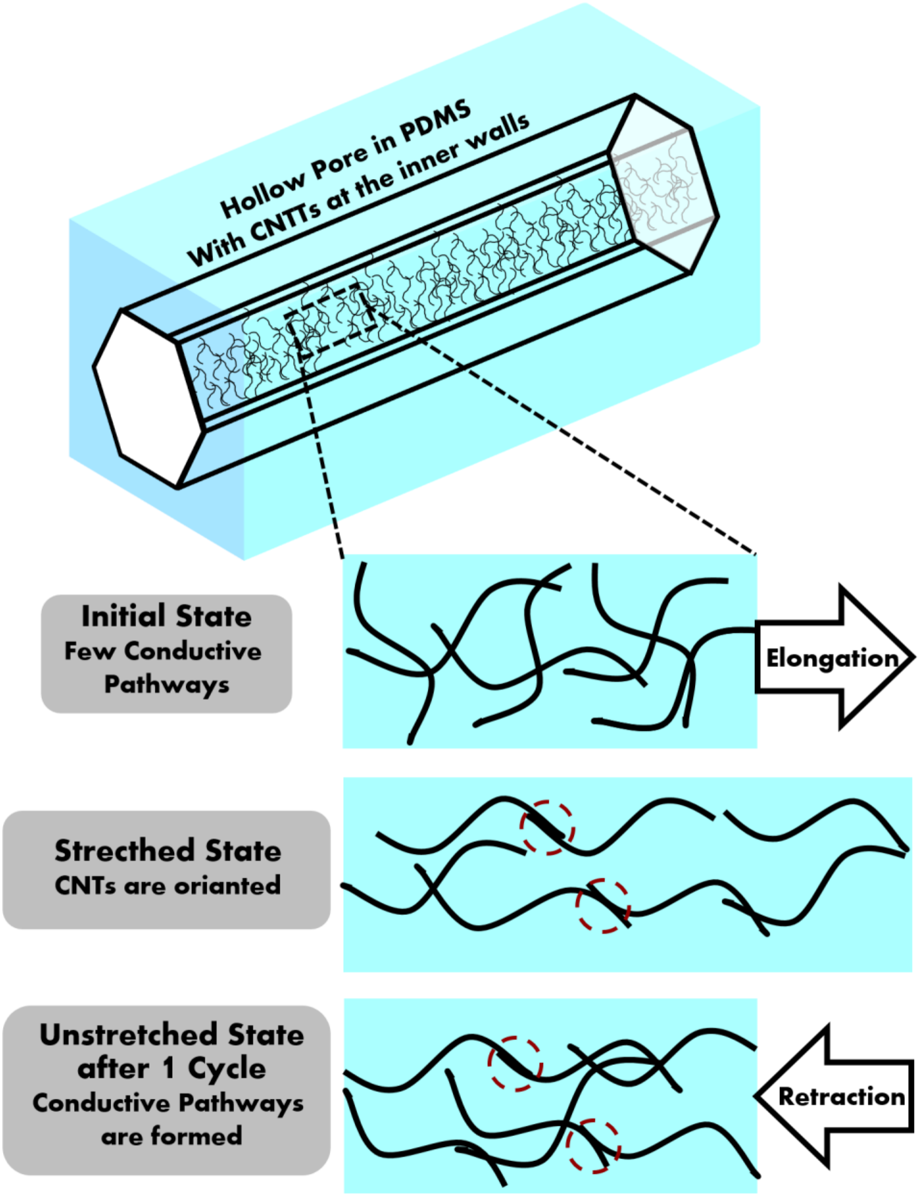

Supplementary Figure 10. Schematic illustration of the self-enhancing effect during cyclic stretching of the CNTT/PDMS composite structures. In the initial state, only a few conductive pathways exist throughout the 3D composite. Upon stretching, the CNTs get unidirectional oriented and aligned in the direction of the applied force. On top of that, some pathways will be broken, leading to a lower conductivity in the stretched state. Thereby the interface area between individual CNTs becomes higher, resulting in large van-der Waals forces (as indicated by the red markers). Upon retraction, some of the CNTs will not recover their original shape, especially at the new formed interconnection points, leading to an increase in conductive pathways.

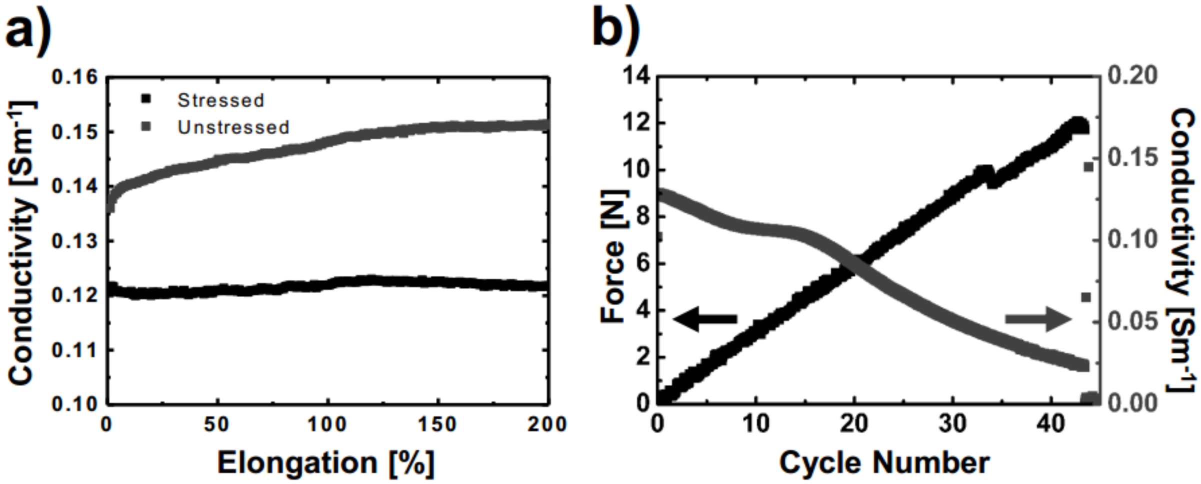

Supplementary Figure 11. Mechanical and electrical properties of a porous stretchable conductor having a carbon nanotube concentration of $1.8x10^{-3}$ g cm^{-3}.

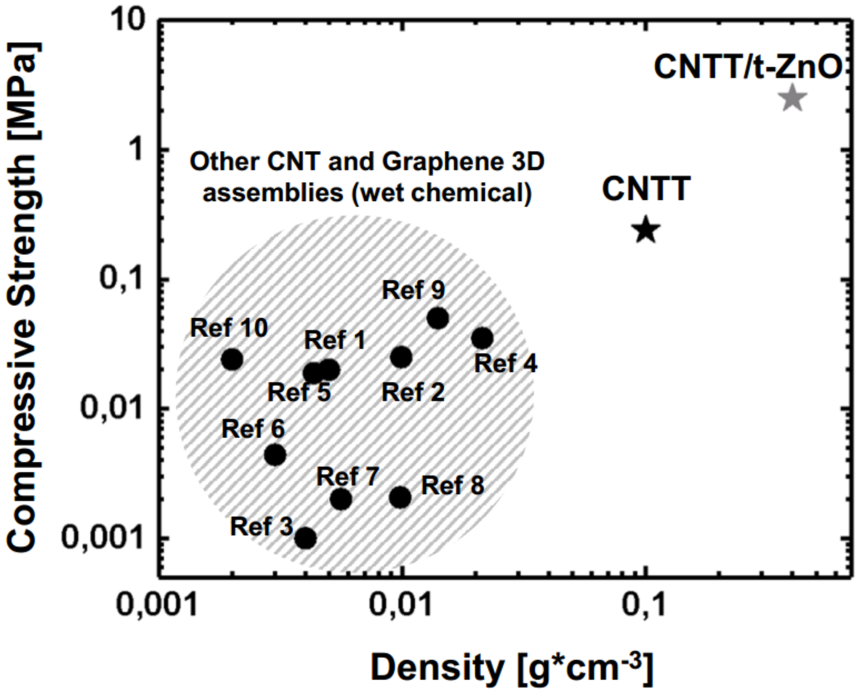

Supplementary Figure 12. Comparison of 3D carbon nanotube and graphene assemblies prepared by wet chemistry[1–10]. The CNTT architecture has a higher mechanical strength under compression.

Supplementary Table

Supplementary Table 1. Mechanical Properties of different CNTT structures

Sample	Denisty (ZnO)	Density (CNT)	Compressive Strength	Specific Compressive Strength	Young's Modulus (Comp.)	Specific Young's Modulus (Comp.)	Tensile Strength	Specific Tensile Strength	Young's Modulus (Tensile)	Specific Young's Modulus (Tensile)
	g/cm³	*g/cm³*	*MPa*	*MPa*cm³/g*	*MPa*	*MPa*cm³/g*	*MPa*	*MPa*cm³/g*	*MPa*	*MPa*cm³/g*
**t-ZnO**	0.300	0.000	0.017	0.057	0.574	1.916	0.007	0.02	0.887	2.957
**t-ZnO/CNTT**	0.300	0.020	0.060	0.186	1.126	3.519	-	-	-	-
**t-ZnO/CNTT**	0.300	0.034	0.203	0.609	4.286	12.832	-	-	-	-
**t-ZnO/CNTT**	0.300	0.047	0.402	1.159	6.678	19.246	-	-	-	-
**t-ZnO/CNTT**	0.300	0.074	1.271	3.397	17.45	46.673	-	-	-	-
**t-ZnO/CNTT**	0.300	0.094	2.539	6.443	24.50	62.185	0.3	0.8	44.88	112.2
**CNTT**	0.000	0.100	0.194	1.940	2.152	21.520	-	-	-	-
**CNTT**	0.000	0.120	0.233	1.942	2.166	18.050	-	-	-	-

Supplementary Discussion

Main factors that govern the formation of the CNT self-entangled structure

1. Template

First of all the template needs to be highly porous, having an open pore structure, with a pore size diameter that is larger compared to the largest dimension of the particle by at least one order of magnitude to allow for the infiltration of particles, thus nano to sub-micro. This is only the case in open pore structures, as the one used in this study. On top of that, this is especially true for nanotubes, due to their large size in one dimension. With increasing template density, the porosity decreases drastically, resulting in smaller pore diameters. In return, this will hinder the formation of a homogeneous coating and thus reinforcement effect, due to the fact, that the CNTs in their dispersed form will not effectively enter into the entire template because of the reduced pore size. Depending on the length of the CNTs a filtering effect will occur, which increases with increasing number of infiltrations. Therefore, higher densities of the template were not considered and 0.3 g cm^{-3} was chosen as the optimum density with a better compromise in terms of its mechanical strength (the pure template is rather soft and thus hard to handle) and porosity (~94%). Thus, a certain pore size (in the µm range) is definitely required to enable a homogenous CNT. However, templates having a density of less than 0.15 g cm^{-3} i.e., with high porosity (>95%), are compressed by the capillary action during CNT infiltration, resulting in a too low mechanical stability of the pure template. They are so fragile that during infiltration, cracks are introduced which lead to a complete collapse of the template's network architecture after several infiltrations.

Another requirement for a homogenous coating (in the case of aqueous dispersions) is the hydrophilicity of the template. Only templates with super-hydrophilic properties will allow for a homogenous coating, since in this case the water spreads over the whole surface due to surface energy minimization. In our case, all these requirements are fulfilled for templates having densities in the range of 0.15 g cm^{-3} to 0.3 g cm^{-3}. Templates having a density lower than 0.15 g cm^{-3} do suffer shrinkage due to their low stability. The influence of the template density on the mechanical properties of the resulting composite materials is shown in Supplementary Figure 5 and discussed in more detail in the following paragraph.

2. Concentration of the used dispersion and kind of nanomaterial

The concentration plays a crucial role when trying to infiltrate the highly porous networks. In most cases, the concentration of the dispersion is limited by the material itself and not by the infiltration process, e.g. how much of the solid can be dispersed

without destabilization. In the case of CNTs, the concentration needs to be adjusted carefully. We have found, that a concentration of around 1 wt% is a good choice for most dispersions. However, this value depends mainly on the dispersion quality and the used CNTs (length, diameter etc.). In general, to high concentrations will increase the formation of agglomerates and nets between the junctions of the template, thereby causing an inhomogeneous coating. To avoid agglomerates, the dispersion should be ultrasonificated before each infiltration step. A lower CNT concentration will form more homogenous layers, however, the infiltration steps will increase to achieve a sufficient coating thickness in terms of mechanical stability, which is unwanted in most cases. On top of that, we observed, that by using CNT dispersions which are low concentrated the entanglement between the individual CNTs will be lower, resulting in a lower reinforcement effect. Every infiltration step will produce a new CNT layer, which is not entangled with the one beneath. Thus, we decided to decrease the concentration to such a level, that we still have a very homogenous coating but the highest degree of entanglement possible with only small deviations in the layer thickness. When using other CNT dispersions, the concentration needs to be adjusted accordingly. Furthermore, the length of the individual CNTs needs to be considered, as mentioned in the description of the template properties.

On top of that, we observed, that the process is self-regulated. After the template is infiltrated several times with CNT dispersion (thus containing a certain amount of CNTs), all the surface is coated at one point. At this point, the structure is not able to take up anymore dispersion and, what is most important: it does not take up any water at all. Therefore, this effect is not related to a self-filtering effect, but can be directly attributed to the shielding of the polarity of the ZnO by the CNT coating, which reduces the hydrophilicity and thus makes it impossible for the template to take up more water. Thereby, the layer thickness can only be enlarged up to a certain point and no complete filling of the template pores is possible. This effect also ensures a widely homogenous coating throughout the 3D structure. Additionally, we observed, that when templates with different densities were infiltrated with the same CNT dispersion, that at lower template densities, this effect started earlier, compared to higher density templates, which can be attributed to the lower surface area that needs to be coated for smaller densities.

For the here presented process we investigated three different commercially available MWCNT dispersions, namely CarboByk 9810 (BYK-Chemie), *CarboDis TN* (Future Carbon), and *Baytubes* DW 55 CM (Bayer MaterialScience). All the experiments and results shown in the main manuscript (and if not state elsewise) were done by using *Baytubes* DW 55 CM. This dispersion was found to give the best results in terms of mechanical reinforcement und structural homogeneity (Supplementary Figure 5). As illustrated in Supplementary Figure 5a, the reinforcement effect is different for the

different CNT dispersions. In the case of CarboDis TN, the effect was even higher compared to the measurements shown in the main manuscript (Baytubes). However, the samples were already saturated at smaller CNT concentrations, resulting in an overall smaller reinforcement effect. On the other hand, using CarboByk, a high amount of CNTs could be infiltrated into the ceramic networks; however, the reinforcement effect was rather low compared to CarboDis TN and Baytubes. We observed, that CarboByk 9810 (diluted to 1 wt%) tends to form a lot of agglomerates next to the coating around the template, resulting in a non uniformity of the films. In the case of CarboDis TN, the CNT coating was very homogenous, similar to the one formed when using *Baytubes* DW 55 CM. A SEM investigation of the homogeneity is shown in Supplementary Figure 2. Therefore, the used CNT dispersion plays a critical role in the reinforcement effect (stabilizing agent, CNT length, CNT diameter etc.). In Supplementary Figure 5b,c,d the effect of the template density is illustrated for different CNT dispersions and concentrations. Form Supplementary Figure 5b it can be observed, that the normalized compressive stress for low CNT concentrations is higher with higher template density. With increasing CNT concentration in the ceramic networks, this trend changes and at high CNT concentrations (0.05 g cm^{-3}) the normalized compressive stress is highest for the lowest template density. This might be directly related to the fact, that in the case of the lower template density, less ZnO surface is available and thus at the same CNT loading, the thickness of the formed self-entangled CNT layer is higher. A similar trend is shown in Supplementary Figure 5c. In contrast to those measurements, when using CarboByk 9810 as the infiltration dispersion, such an effect is not observed. The highest template density has always the highest normalized compressive stress. Again, this is related to the high amount of agglomerates formed when using this dispersion. Furthermore in Supplementary Figure 5e,f the effect of CNT concentration in the dispersion is illustrated. There is no change in the case of CarboByk (Supplementary Figure 5f), which is again a result of the inhomogeneous coating process and high amount of agglomerates. In the case of CarboDis TN there is a big difference between templates coated with a 0.5 wt% or 1 wt% dispersion. At first glance, this effect is unexpected: By infiltrating the ceramic template with a low wt% CNT dispersion one might expect to form a more homogenous coating compared to a higher concentrated one. Even though this might be the case, the self-entanglement is much higher in the case of a higher CNT concentration in the dispersion. In other words, when we have one self-entangled CNT layer on a substrate (dried state) and we apply another CNT coating. There is no entanglement between those two layers. However, if we directly use a higher concentrated CNT dispersion to form a film of same thickness, the self-entanglement will be the same throughout the whole film, thus leading to higher mechanical properties.

(Please note: a special designed CNT dispersion might lead to a much higher effect.)

3. Infiltration methods

As by our observations, the infiltration method itself has no influence on the formed structure. As shown in our supporting video S1, the sample is infiltrated from the top (as it was the case for all samples for this manuscript). However, this was simply done for simplicity of the process. Due to the large capillary forces and the hydrophilicity of the template, it is also possible to infiltrate the template from every side or just by placing it on a drop of dispersion. However, this has no effect on the formed CNTT assembly. Furthermore, the temperature at which the samples are dried has an influence. Higher temperatures lead to faster water evaporation and thus to a more inhomogeneous self-assembly of the CNTs. However, at low temperatures, the water will only slowly evaporate and even some residues might stay in the network, which might hinder the complete filling in the next infiltration step. We found, that temperatures of around 50 °C lead to better results in terms of CNT self-assembly.

Supplementary References

1. Dong, L. *et al.* Facile preparation of carbon nanotube aerogels with controlled hierarchical microstructures and versatile performance. *Carbon* **90,** 164–171 (2015).
2. Hu, H., Zhao, Z., Wan, W., Gogotsi, Y. & Qiu, J. Ultralight and highly compressible graphene aerogels. *Advanced Materials* **25,** 2219–2223 (2013).
3. Kohlmeyer, R. R., Lor, M., Deng, J., Liu, H. & Chen, J. Preparation of stable carbon nanotube aerogels with high electrical conductivity and porosity. *Carbon* **49,** 2352–2361 (2011).
4. Xu, Y., Sheng, K., Li, C. & Shi, G. Self-assembled graphene hydrogel via a one-step hydrothermal process. *ACS Nano* **4,** 4324–4330 (2010).
5. Kim, K. H., Oh, Y. & Islam, M. F. Graphene coating makes carbon nanotube aerogels superelastic and resistant to fatigue. *Nat. Nanotechnol.* **7,** 562–566 (2012).
6. Kim, K. H., Oh, Y. & Islam, M. F. Mechanical and Thermal Management Characteristics of Ultrahigh Surface Area Single-Walled Carbon Nanotube Aerogels. *Advanced Functional Materials* **23,** 377–383 (2013).
7. Sun, H., Xu, Z. & Gao, C. Multifunctional, ultra-flyweight, synergistically assembled carbon aerogels. *Advanced Materials* **25,** 2554–2560 (2013).
8. Zou, J. *et al.* Ultralight multiwalled carbon nanotube aerogel. *ACS Nano* **4,** 7293–7302 (2010).
9. Bryning, M. B. *et al.* Carbon Nanotube Aerogels. *Advanced Materials* **19,** 661–664 (2007).
10. Peng, Q. *et al.* Graphene nanoribbon aerogels unzipped from carbon nanotube sponges. *Adv. Mater.* **26,** 3241–3247 (2014).
