# Supplementary material for: Hierarchical self-entangled carbon nanotube tube networks
Source: Nat Commun. 2017 Oct 31;8:1215. doi: 10.1038/s41467-017-01324-7 (PMC5662747; doi:10.1038/s41467-017-01324-7)
Supplement: Supplementary file 2 — Description of Additional Supplementary Files [file 41467_2017_1324_MOESM2_ESM.docx]

**Description of Additional Supplementary Files**

**File name:** Supplementary Movie 1

**Description:** 3D ceramic templates are infiltrated with an aqueous carbon nanotube dispersion using a custom designed computer controlled syringe setup and dried under ambient conditions on a hot plate (40 °C). During infiltration, the aqueous carbon nanotube dispersion is maximizing its interface area because of the hydrophilic surface of the template. When the first drop of CNT dispersion gets in contact with the ceramic template, it will penetrate a certain volume of the template, namely the volume that is needed to maximize its surface area, because of the hydrophilic properties of the template. Following droplets will increase this volume (more surface area is needed). Thus, at some point the template is completely filled with dispersion and increasing the amount will lead to the formation of a drop forming on top of the template surface. Using a template with a density of 0.3 g cm^-^³ (94% porosity) and a volume of 0.17 cm³ the free volume is equal to around 0.16 cm³. Thus, the template should be able to take up 160 µl of water before a droplet forms on the top surface, which indeed is the case. Therefore, after infiltration, the whole network is flooded and due to the high porosity of the network, the CNT concentration can be assumed to be constant all over the sample.

Furthermore, it has to be pointed out, that the flow rate of the CNT dispersion through the template seems to be non-homogenous. Indeed, it is very homogenous but appears to be different due to very tiny variations in the pore sizes of the ceramic template. Pores, which are a little closer provide higher capillary forces and are thus chosen to be wetted first by the CNT dispersion. Subsequent liquid droplets wet homogenous the non-wetted areas. This is also shown by the fact, that there is no filtering effect visible. After complete infiltration, the sample has a homogenous color (there is no black area on top, which gets greyer when moving down).

(Note: The Video shows only the first infiltration in real time).

**File name:** Supplementary Movie 2

**Description:** As was shown in Video S1, the ZnO template is infiltrated with a 0.5 wt% CNT dispersion for one time (complete infiltration). Afterwards, the infiltrated sample is moved directly (no drying time) into a beaker filled with distilled water. Upon water contact, the CNT dispersion in the template is not mixing with the surrounding water. Only a small amount of CNTs are removed from the template, even when the mixture is stirred. As a comparison, if the same amount of CNT dispersion (as was added to the pure template) is mixed with the water, the color change is very strong. This proves the fact, that there is some certain interaction between the CNTs and the ZnO template, though which the CNTs get distributed homogenously on the ZnO surfaces.

**File name:** Supplementary Movie 3

**Description:** Compression tests on a reference 3D ceramic template (right) and a template containing 0.1 g/cm³ carbon nanotubes (left). After the force is removed, the carbon nanotube containing structure shows a higher restoring force compared to the pure template, thereby indicating that individual carbon nanotubes in the CNTT structure get employed in their tensile properties, even though the sample is compressed.

**File name:** Supplementary Movie 4

**Description:** Compression tests on a reference 3D CNTT network of 0.10 g cm^-^³ carbon nanotubes. The load is applied along the vertical directions in the video. Contour plot of the elastic strain is shown. It clearly appears how the tubes are employed in bending and in tension under a compressive load on the specimen. Analogously to aerographite tetrapodal networks previously studied by the authors, the deformation behavior of the entangled CNTT network is governed by the formation of buckling hinges at tetrapod joints or within the arms. The latter are prone to appear in the proximity in the entangling joints between different tetrapods.
